# Three mechanisms control E-cadherin localization to the zonula adherens

**DOI:** 10.1038/ncomms10834

**Published:** 2016-03-10

**Authors:** Innokenty Woichansky, Carlo Antonio Beretta, Nicola Berns, Veit Riechmann

**Affiliations:** 1 Department of Cell and Molecular Biology and Division of Signaling and Functional Genomics at the German Cancer Research Center (DKFZ), Medical Faculty Mannheim, Heidelberg University, Ludolf-Krehl-Strasse 13-17, D-68167 Mannheim, Germany; 2 Heidelberg University, COS and Nikon Imaging Center at the University of Heidelberg, Bioquant, D-69120 Heidelberg, Germany; 3 Excellenzcluster CellNetworks, University of Heidelberg, D-69120 Heidelberg, Germany

## Abstract

E-cadherin localization to the zonula adherens is fundamental for epithelial differentiation but the mechanisms controlling localization are unclear. Using the *Drosophila* follicular epithelium we genetically dissect E-cadherin transport in an *in vivo* model. We distinguish three mechanisms mediating E-cadherin accumulation at the zonula adherens. Two membrane trafficking pathways deliver newly synthesized E-cadherin to the plasma membrane. One is Rab11 dependent and targets E-cadherin directly to the zonula adherens, while the other transports E-cadherin to the lateral membrane. Lateral E-cadherin reaches the zonula adherens by endocytosis and targeted recycling. We show that this pathway is dependent on RabX1, which provides a functional link between early and recycling endosomes. Moreover, we show that lateral E-cadherin is transported to the zonula adherens by an apically directed flow within the plasma membrane. Differential activation of these pathways could facilitate cell shape changes during morphogenesis, while their misregulation compromises cell adhesion and tissue architecture in differentiated epithelia.

## Abstract (web summary)

E-cadherin is an adhesion molecule mediating cell-cell adhesion; correct localization is important but how localization is controlled is not clear. Here the authors use *Drosophila* as a model system to distinguish three distinct trafficking pathways that direct E-cadherin to the zonula adherens.

## Graphical abstract

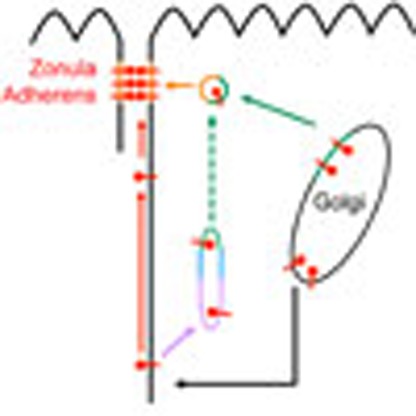

Adherens junctions (AJs) are cell–cell contacts that mediate intercellular adhesion of epithelial cells. They control tissue architecture by regulating cell shape and adhesion. AJs are formed by the homophilic adhesion receptor E-cadherin (E-cad), whose cytoplasmic domain is associated with several proteins. Among them are β- and α-catenin, which anchor the actin cytoskeleton at the plasma membrane (PM)^1^,^2^,^3^. Changes in E-cad distribution at the PM lead to severe tissue disorders that underlie many disease processes. For instance, loss of E-cad in tumour cells releases AJ-mediated cell–cell contacts, which is a critical first step during metastasis^4^,^5^,^6^.

AJs form the zonula adherens (ZA), an adhesive belt that links epithelial cells into a continuous sheet. In *Drosophila* the ZA is located in the most apical region of the lateral membrane, where it defines the border between the lateral and the apical membrane domain. AJ components also localize along the lateral PM, where they have a more punctate pattern compared with the continuous ZA. It has been shown in mammalian cells that punctate E-cad clusters establish the lateral actin cortex^7^.

AJ components are constantly internalized from and recycled back to the PM^8^, a trafficking process that allows tissue remodelling during development. Many morphogenetic processes including gastrulation or epithelial mesenchymal transitions rely on controlled AJ disassembly, whereas the formation of new epithelia during mesenchymal epithelial transitions (MET) requires AJ assembly at defined cell–cell contact sites^2^,^6^,^9^,^10^. Furthermore, AJ trafficking is required for epithelial maintenance, and disrupting AJ trafficking affects ZA maintenance and may eventually lead to loss of epithelial adhesion^11^,^12^,^13^,^14^.

E-cad trafficking occurs in membrane vesicles whose formation, transport and targeting is controlled by Rab GTPases^15^,^16^,^17^. Rab5 is central for the formation of the early endosome to which endocytosed vesicles are delivered. Within the early-endosome internalized proteins are sorted either for degradation in the endolysosomal pathway or for recycling. Recycling to the PM can occur either in a direct and rapid pathway controlled by Rab4 or via the recycling endosome, which involves Rab11 (ref. 18).

E-cad protein is translated at the ER and membrane vesicles with newly synthesized protein are sorted at the Golgi for transport to the lateral PM. Sorting in vertebrate cells requires a dileucine motif in the cytoplasmic tail of E-cad^19^, which is, however, not present in the *Drosophila* orthologue. Transport of E-cad vesicles to the lateral PM involves the passage through a Rab11-positive compartment^20^.

A recycling mechanism redistributes E-cad from the lateral PM to the apicolateral PM, which leads to E-cad accumulation at the ZA. Recycling involves molecular interactions between Rab11, the exocyst complex and β-catenin^11^. The exocyst complex is thought to provide a landmark on the PM, which targets the fusion of vesicles^21^,^22^. Targeted E-cad recycling is so far the only identified mechanism explaining the accumulation of AJ components at the ZA. Here we identify two additional mechanisms that localize E-cad to the ZA: direct transport of E-cad vesicles from the Golgi to the ZA and an actin driven and apically directed cadherin flow within the lateral PM. Moreover, we identify with RabX1 a critical new component of the recycling pathway, which links the early and the recycling endosome.

## Results

### *Rab5* and *Rab11* differentially affect DE-cad distribution

The *Drosophila* ovary consists of egg chambers (or follicles) which form when epithelial precursors migrate towards a cyst of germline cells (pink cells Supplementary Fig. 1a). When they reach the cyst they undergo a MET including ZA formation and PM polarization^23^,^24^. Approximately 30 cuboidal epithelial cells assemble an epithelial monolayer around the cyst when a new egg chamber is formed^25^. During early oogenesis stages epithelial cells proliferate, but at mid-oogenesis cell division ceases and morphogenesis starts^26^.

In a genome scale *in vivo* RNA interference (RNAi) screen for genes involved in the formation of the follicular epithelium we identified strong epithelial defects after *Rab5* and *Rab11* knockdown^27^. To examine whether the two Rab GTPases contribute to *Drosophila* E-cadherin (DE-cad) trafficking we analysed the localization of Armadillo (Arm, *Drosophila* β-catenin), which binds DE-cad not only at the PM but also during vesicular transport^11^,^20^,^28^. In wild-type epithelia Arm is detectable along the lateral PM and concentrates apicolaterally at the ZA (Supplementary Fig. 1b,b′). After *Rab5* depletion by RNAi this apicolateral concentration is severely reduced and Arm accumulates uniformly along the lateral PM (Supplementary Fig. 1c,c′; Supplementary Table 1, which summarizes the reproducibility of all RNAi experiments). This is in striking contrast to *Rab11* RNAi, which leads to strong Arm accumulation within the cell (Supplementary Fig. 1e,e′, see also ref. 29).

**Figure 1:**
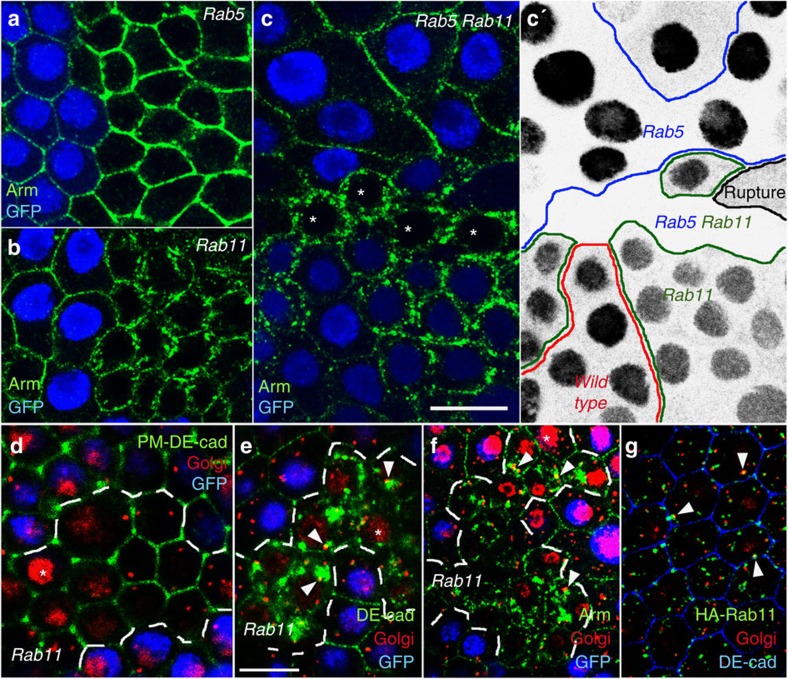
*Rab11* mutants accumulate *de novo* synthesized DE-cad. Optical confocal sections perpendicular to the apico-basal axis of the follicular epithelium. (**a**–**f**) Show genetic mosaics in which homozygous mutant cells are indicated by the absence of GFP (blue) (**a**) *Rab5* mutant cells accumulate Arm at the cell periphery. (**b**) *Rab11* mutant cells accumulate Arm within the cell. (**c**) *Rab5 Rab11* double-mutant cells (asterisks) accumulate Arm like *Rab11* single mutants within the cell. (**c**′) *Rab5* and *Rab11* are located on two different chromosomes and therefore clone induction resulted in *Rab5* single, *Rab11* single and *Rab5 Rab11* double-mutant cells. The genotype of each cell can be identified by the GFP signal shown in black. *Rab5* single-mutant cells lose cytoplasmic GFP, *Rab11* mutant cells lose strong nuclear GFP and double-mutant cells express no GFP. Cell clone boundaries are marked. (**d**) Living ovaries harbouring *Rab11* mutant cells were incubated for three hours with an anti-DE-cad antibody to label DE-cad at the PM and to allow its endocytosis. After washing, fixation and PM permeabilization DE-cad (green) was detected and ovaries were counterstained for the Golgi marker GM130 (red). No intracellular accumulation of DE-cad is detectable. Note that the anti-GM130 antibody gives an unspecific signal in the nucleus (asterisks). (**e**) DE-cad detection in Rab11 clones after PM permeabilisation reveals accumulation of intracellular DE-cad aggregates, which partially overlap with the Golgi (arrowheads). (**f**) *Rab11* clone stained for the Golgi marker GM130 and Arm. Arrowheads mark sites where Arm aggregates about the Golgi. (**g**) *traffic jam*-Gal4 induced HA-Rab11 protein (green) localizes close to and partially overlaps with the Golgi (red). Scale bar, 10 μm.

We validated the RNAi phenotypes by generating genetic mosaics, which allowed us to directly compare Arm distribution in mutant and neighbouring wild-type cells. Optical confocal sections through the lateral PM reveal a strong Arm and DE-cad accumulation at the periphery of *Rab5* mutant cells (Fig. 1a, Supplementary Fig. 1d and Supplementary Table 2, which summarizes the reproducibility of all experiments with genetic mosaics). Ultrastructural analysis in *Drosophila* ooyctes revealed that loss of *Rab5* leads to the accumulation of endocytic vesicles close to the PM^30^, and it appears therefore likely that the enlarged Arm domain reflects not only the PM but also endocytosed protein near the PM. In *Rab11* cells Arm and DE-cad are only weakly detectable at the lateral membrane and accumulate in numerous aggregates within the cell (Fig. 1b,e). Bigger *Rab11* mutant cell clones show no clear ZA and flatten (yellow arrows in Supplementary Fig. 1f). This suggests that the intracellular accumulation of DE-cad in *Rab11* mutant cells results in decreased DE-cad levels at the PM, which lead to cell shape and cell–cell adhesion defects.

### Rab11 regulated exocytosis transports DE-cad to the PM

DE-cad is endocytosed at the lateral PM, and Rab11 is involved in a targeted recycling process that leads to DE-cad accumulation at the ZA^11^. To test if the intracellular DE-cad aggregation in *Rab11* cells is caused by a recycling failure we prevented DE-cad influx from the PM by generating *Rab5 Rab11* double-mutant cells. If the intracellular DE-cad aggregates in *Rab11* are formed by endocytosed DE-cad, the block of early-endosome formation in the double mutants would hold internalized DE-cad at the periphery and intracellular DE-cad aggregation would be suppressed. However, *Rab5 Rab11* double-mutant cells did not affect intracellular DE-cad accumulation arguing against a recycling defect (asterisks in Fig. 1c). We also knocked down *Rab5* and *Rab11* simultaneously (Supplementary Fig. 1g,g′) using two RNAi lines that phenocopy the null phenotypes (Supplementary Fig. 1c,e). The double knockdown shows the same intracellular accumulation like the *Rab11* single RNAi confirming that a block of early-endosome formation in *Rab11* does not prevent intracellular DE-cad aggregation. These data indicate that a large part of the intracellular DE-cad protein in *Rab11* cells does not come from the PM, and hence its aggregation is not primarily caused by a recycling defect.

To examine a possible contribution of endocytosed DE-cad to the intracellular aggregates we performed endocytosis experiments. We incubated living ovaries harbouring *Rab11* clones with an antibody that binds to the extracellular part of DE-cad^31^,^32^. As the PM was not permeabilized during the incubation, the antibody bound exclusively to DE-cad at the PM (PM-labelled DE-cad) but not to intracellular protein. Studies in cell-free systems suggest that antibody binding inhibits trans interactions between E-cad molecules and promotes their endocytosis^33^. After 3-h incubation we fixed the ovaries, permeabilized the PM and detected DE-cad. We could not identify intracellular DE-cad aggregates in these assays supporting the idea that endocytosed DE-cad does not contribute to the intracellular accumulation (Fig. 1d). We therefore conclude that the intracellular aggregates consist mainly of *de novo*-synthesized DE-cad.

Considering the well-established function of Rab11 in recycling, it is unexpected that endocytosed DE-cad does not accumulate at all. A possible explanation is that in the absence of Rab11 all endocytosed DE-cad is directly recycled back to the PM. Rab4, the regulator of direct recycling, might mediate DE-cad recycling partially in wild-type cells and completely if Rab11 is absent. Consistent with this hypothesis we detected cytoplasmic Arm puncta that co-localize with Rab4 in wild-type and *Rab11* cells (Supplementary Fig. 2a).

Our data suggest that the intracellular aggregates in *Rab11* cells do not consist of recycled but rather of newly synthesized DE-cad protein. New DE-cad is translated at the ER and its exocytosis to the PM involves passage through the Golgi. This organelle consists of several transitional ER-Golgi subunits in *Drosophila*^34^. Notably, the aggregates within *Rab11* cells directly abut the Golgi (Fig. 1e,f) supporting the idea that after leaving the Golgi, vesicles with newly synthesized DE-cad protein accumulate within the cell.

To examine whether Rab11 localizes to the Golgi in wild-type cells we expressed HA-tagged Rab11 protein in the follicular epithelium. Quantification of the overlap of Rab11 with the Golgi marker GM130 revealed that 63% of the detected Golgi puncta (*n*=1211) overlapped with HA-Rab11 puncta (*n*=4811). This concentration of Rab11 at the Golgi is consistent with a role of Rab11 in regulating exocytosis. Collectively, these data suggest that Rab11 controls the transport of newly synthesized DE-cad from the Golgi to the PM.

### *Rab11* controls DE-cad exocytosis to the apicolateral PM

**Figure 2:**
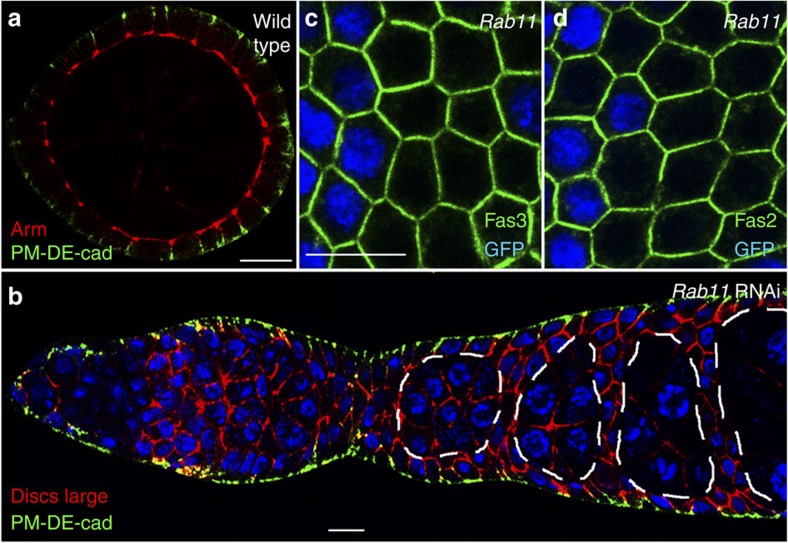
DE-cad exocytosis to the lateral PM occurs in the absence of *Rab11*. (**a**) Sagittal optical section through a wild-type egg chamber showing the follicular epithelium along its apical–basal axis. Living ovaries were briefly incubated with an anti-DE-cad antibody, washed, fixed and counterstained for Arm to detect the ZA (red). DE-cad (green) is only detectable in the basal region of the lateral PM. (**b**) Sagittal optical section through an ovariole in which Rab11 was depleted by RNAi. Ovaries were briefly incubated with the anti-DE-cad antibody and counterstained for Discs large protein (red), which localizes to the lateral PM of the epithelium and to the membranes of the germline cells. Older germline cysts are encircled by the dashed line. DE-cad (green) is only detectable in the basal part of the lateral PM. (**c**,**d**) Optical confocal sections perpendicular to the apico-basal axis of the follicular epithelium showing *Rab11* mutant clones stained for Fas3 and Fas2 (green). The localization of both proteins is not affected. Scale bar, 10 μm.

DE-cad localizes to the ZA and to the lateral PM. *De novo*-synthesized DE-cad could therefore be transported by apicolaterally and laterally directed pathways. To examine the lateral pathway we incubated living ovaries only briefly with the DE-cad antibody to avoid DE-cad endocytosis and redistribution. After short incubation of wild-type ovaries, DE-cad was exclusively detectable in the basal region of the lateral PM, but not at the ZA (Fig. 2a). Septate junctions, the equivalent of tight junctions in insect cells, could prevent access of the antibody to the ZA. However, ultrastructural studies^35^ and analysis of the dynamics of septate junction components^36^ argue against the existence of functional septate junctions at early and mid-oogenesis stages. We therefore speculate that the incubation time in this experiment is too short to allow the antibody to reach the ZA.

To test whether Rab 11 is required for DE-cad exocytosis to the lateral PM we knocked down *Rab11* in all epithelial cells including their precursors. We achieved this by using *traffic jam*-Gal4, which strongly induces RNAi in all somatic cells^37^. After brief incubation of these ovaries with the DE-cad antibody, DE-cad was clearly detectable at the basolateral PM (Fig. 2b). This indicates that DE-cad is exocytosed to the lateral PM in the absence of Rab11. Thus, *Rab11* depleted cells accumulate newly synthesized DE-cad within the cell but show no defects in basolateral exocytosis. We therefore conclude that the accumulating DE-cad protein is destined for apicolateral exocytosis to the ZA.

Exocytosis to the lateral PM might be mediated by a pathway that is common for all lateral membrane proteins. To further investigate a possible role of Rab11 in this pathway we examined the localization of the lateral adhesion proteins Fasciclin2 (Fas2) and Fasciclin3 (Fas3). Both proteins localize normally and reveal no intracellular aggregation in *Rab11* mutant cells (Fig. 2c,d). Thus, Rab11 is not essential for exocytosis of adhesion proteins to the lateral PM. In summary, our data indicate the existence of two exocytosis pathways to the lateral PM: a Rab11-independent pathway for exocytosis to the basal region of the lateral PM and a Rab11-dependent pathway that targets exocytosis to the ZA.

It has been shown that Rab11 recruits the exocyst complex to endocytosed DE-cad vesicles to promote their fusion with the ZA^11^. To examine whether Rab11 also cooperates with the exocyst complex in targeting newly synthesized DE-cad to the ZA we analysed Sec6, a critical exocyst component. The aggregating AJ components in *Rab11* mutant cells show only a very weak overlap with Sec6 protein (Supplementary Fig. 2b). This is consistent with the idea that Rab11 is required to recruit the exocyst complex to vesicles with newly synthesized DE-cad. *Sec6* mutant cells form intracellular Arm aggregates resembling the aggregates in *Rab11* mutant cells. These Arm aggregates also accumulate Rab11 protein. This suggests that AJ components are trapped in a Rab11 compartment when exocyst function is absent (Supplementary Fig. 2c). Thus, our data support a model in which the Rab11-exocyst interaction regulates the targeting of vesicles with endocytosed as well as newly synthesized DE-cad. We therefore propose a new exocytosis pathway, in which Rab11 and the exocyst complex guide *de novo* synthesized DE-cad to the apicolateral PM.

### Loss of *RabX1* leads to intracellular DE-cad aggregation

**Figure 3:**
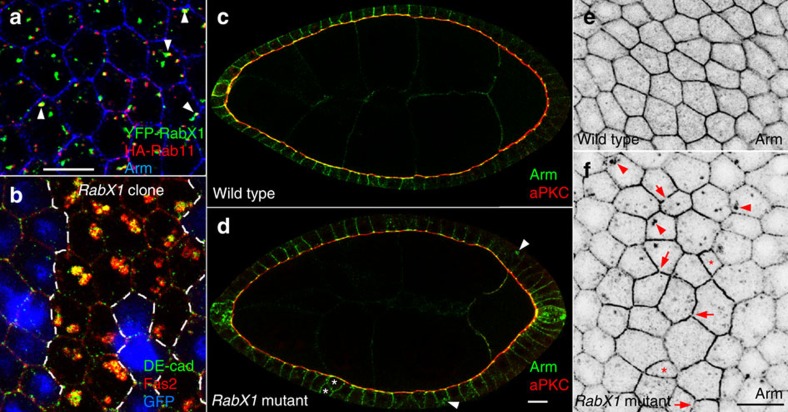
*RabX1* mutants show cell shape defects and intracellular accumulation of cell adhesion proteins. (**a**,**b**) Optical confocal sections perpendicular to the apico-basal axis of the follicular epithelium. (**a**) *traffic jam*-Gal4 induced YPF-RabX1 (green) and HA-Rab11 (red) proteins overlap (arrowheads) in wild-type cells. (**b**) *RabX1* mutant cell clone marked by the absence of GFP (blue) stained for DE-cad (green) and Fas2 (red). The yellow overlap of the signals indicates that the two proteins accumulate within the same compartment. (**c**,**d**) Sagittal optical section through egg chambers comparing wild-type (**c**) and homozygous *RabX1* mutant (**d**) egg chambers stained for the apical marker aPKC (red) and Arm (green). *RabX1* mutants localize aPKC normally but show cell shape defects (asterisk) and intracellular Arm aggregation (arrowheads). (**e**,**f**) Optical sections perpendicular to the apical–basal axis comparing wild-type and homozygous mutant *RabX1* epithelia stained for Arm. (**f**) In *RabX1* mutants some epithelial cells show shape abnormalities (asterisks), the ZA reveals gaps (arrows) and Arm accumulates within the cells (arrowheads). Scale bar, 10 μm.

To identify new Rab GTPases controlling DE-cad trafficking we focused on Rab proteins that co-localize with Rab11 in *Drosophila* neuronal cells^38^. We found that 31% of the Rab11 vesicles (*n*=4,206) in the follicular epithelium overlap with vesicles of the so far uncharacterised GTPase RabX1 (*n*=1,586, Fig. 3a). To analyse *RabX1* function we used a P-element insertion into the 5´UTR (*RabX1*^*KG06805*^). After removal of second site mutations homozygous flies were viable (see Methods). Mutant epithelia polarize their membrane domains normally but show cell-shape defects (Fig. 3c–f). Quantitative analysis of confocal pictures revealed a strong reduction of hexagonal cells, which is the predominant shape in the wild-type (Table 1). Moreover, measurements of cell areas show an increased average cell size, which is, however, accompanied by a greater variability in size (compare s.d. in Table 1). Although Arm and DE-cad localize to the lateral PM and accumulate apicolaterally in *RabX1* mutants, the formation of a continuous ZA is affected (arrows in Fig. 3f). We observed these ZA discontinuities in 46% of mutant cells (*n*=114), while they were detectable only in 19% of wild-type cells (*n*=118). Remarkably, *RabX1* mutant epithelia also show big intracellular Arm aggregates indicating a defect in DE-cad trafficking (arrowheads in Fig. 3d,f). Interestingly, intracellular aggregation is not restricted to AJ components since DE-cad accumulates together with Fas2 (Fig. 3b, 84% of the analysed DE-cad aggregates (*n*=74) also accumulated Fas2). This suggests that DE-cad and Fas2 accumulate in the same compartment.

*RabX1*^*KG06805*^ is viable over a deficiency (Df(2R)BSC661), and hemizygous egg chambers show the same morphological defects and intracellular aggregates like homozygous mutants (Supplementary Fig. 3a,b). This indicates that *RabX1*^*KG06805*^ is either a null or a strong allele. Importantly, expression of a UAS-*YFP-RabX1* transgene completely rescues all morphological defects as well as the intracellular aggregation of Arm and Fas2 (Supplementary Fig. 3c–f and Supplementary Table 2) confirming that the epithelial defects are caused by the mutation in *RabX1*.

### *RabX1* links early and recycling endosomes

The intracellular aggregation of membrane proteins in *RabX1* mutants could reflect defects in endolysosomal degradation, exocytosis or recycling. Our data argue against the first two possibilities. Defects in the endolysosomal pathway caused by mutations in *Rab5* or ESCRT components result in overproliferation. This leads to the formation of elongated cellular connections between egg chambers (see Supplementary Fig. 1c for *Rab5*, arrowheads) and multi-layered epithelia^27^,^39^,^40^. *RabX1* mutants do not show these overproliferation phenotypes suggesting that the gene does not affect the endolysosomal pathway.

To examine whether exocytosis to the lateral PM is *RabX1* dependent we briefly incubated living ovaries of mutants with the DE-cad antibody, and detected the protein at the basolateral PM (Supplementary Fig. 4a). This indicates that RabX1 is not essential for lateral DE-cad exocytosis.

To access whether RabX1 is, like Rab11, involved in DE-cad exocytosis to the ZA we compared the pattern of Arm aggregation in *RabX1* and *Rab11* mutant cells by quantifying confocal images depicting Arm and Golgi localization (see Fig. 1f and Supplementary Fig. 4b for examples). This revealed a seven times higher frequency of Arm–Golgi overlaps in *Rab11* cells (Supplementary Table 3). Moreover, in *Rab11* cells the average size of Arm aggregates is smaller and their total number higher. Thus, *Rab11* cells generate numerous small aggregates, whereas *RabX1* cells form few aggregates which are larger. These differences in Arm aggregation suggest that Rab11 and RabX1 affect different DE-cad localization pathways, and thus argue against a role of RabX1 in apicolateral exocytosis.

**Figure 4:**
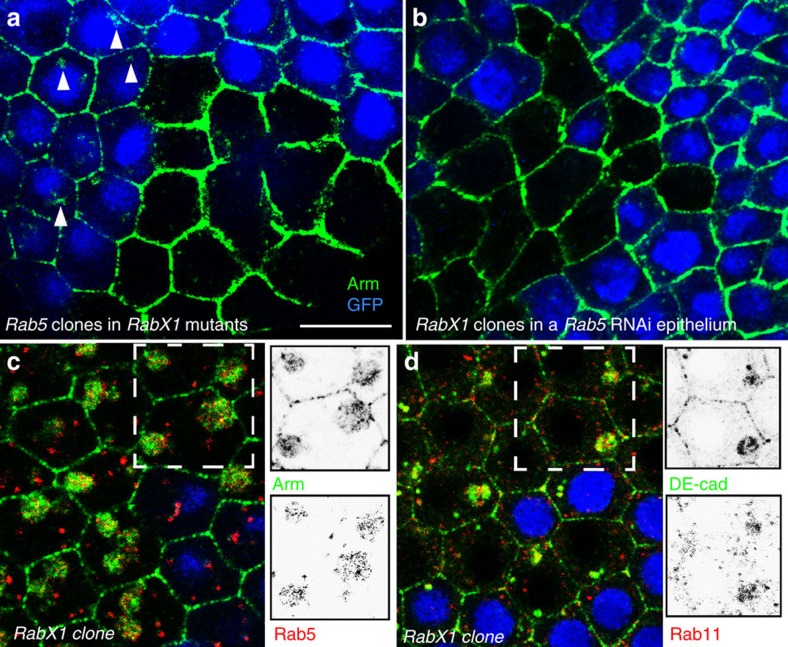
*RabX1* acts downstream of *Rab5* and prevents the intracellular aggregation of Arm, DE-cad, Rab5 and Rab11. Optical confocal sections perpendicular to the apico-basal axis of the follicular epithelium are shown. (**a**) *Rab5* mutant cell clone induced in homozygous *RabX1* mutant females marked by the absence of GFP (blue). Arm (green) aggregates form in *RabX1* mutant *Rab5* wild-type cells (arrowheads), whereas *RabX1 Rab5* double-mutant cells accumulate Arm at the cell periphery. (**b**) *RabX1* mutant cell clones (indicated by loss of GFP (blue)) were induced in epithelia with GR1-Gal4 induced *Rab5* RNAi. Arm (green) aggregates are not formed in *RabX1* cells when *Rab5* is depleted. (**c**,**d**) *RabX1* mutant cell clones marked by the absence of GFP (blue). Insets show single channels of the region marked by the white frame. (**c**) Rab5 protein (red) accumulates within the Arm aggregates (green). (**d**) Rab11 protein (red) accumulates within the DE-cad aggregates (green). Scale bar, 10 μm.

To test whether *RabX1* acts in recycling we prevented the DE-cad influx from the PM in homozygous *RabX1* mutants by inducing *Rab5* clones. This led to an accumulation of Arm at the periphery of double-mutant cells, which was accompanied by a strong reduction or disappearance of the intracellular Arm aggregates (Fig. 4a). We also induced *RabX1* clones in epithelia with *Rab5* knockdown, which again suppressed aggregation (Fig. 4b). Thus, the formation of intracellular DE-cad aggregates in *RabX1* is *Rab5* dependent and occurs downstream of early-endosome formation.

To further characterize the intracellular DE-cad aggregates in *RabX1* cells we analysed whether they co-localize with Rab5 and Rab11. Remarkably, both Rab GTPases concentrated within the aggregates (Fig. 4c,d; 94% of Arm aggregates (*n*=402) accumulated Rab5 and 95% DE-cad aggregates (*n*=180) accumulated Rab11). Localization of Rab5 and Rab11 was also examined by expressing HA-tagged Rab5 and Rab11 in *RabX1* mutants, and confirmed that the two Rab proteins co-accumulate with Arm (Supplementary Fig. 4c,d). In summary, these data suggest that in *RabX1* cells endocytosed DE-cad is trapped in a large intracellular compartment, which accumulates regulators of the early and the recycling endosome. Notably, in wild-type cells RabX1 protein strongly overlaps with Rab11 (Fig. 3a, 83% of the analysed RabX1 puncta (*n*=1,586) overlapped with Rab11) and Rab5 (Supplementary Fig. 4e, 83% RabX1 puncta (*n*=829) overlapped with Rab5). This supports the idea that RabX1 provides a direct link between the early and the recycling endosome.

**Figure 5:**
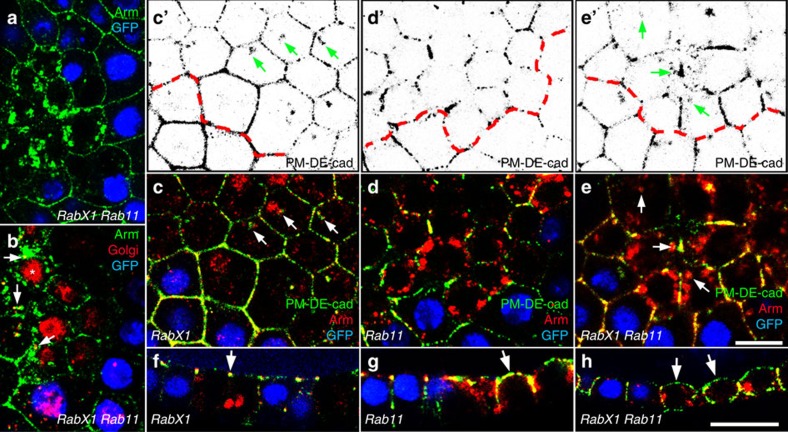
*RabX1 Rab11* double mutants accumulate *de novo* synthesized as well as endocytosed DE-cad. (**a**,**b**) *Rab11* mutant cell clones induced in homozygous *RabX1* mutants. (**a**) Double-mutant cells (recognized by the absence of GFP (blue)) accumulate Arm (green) in numerous small aggregates within the cell. (**b**) Aggregates partially overlap with the Golgi (red, arrows). Asterisks marks nuclear background signal of the Golgi marker. (**c**–**f**) Endocytosis assays in which ovaries were incubated with an anti-DE-cad antibody for 3 h. PM-labelled DE-cad is shown in green. Arm labelling (red) was performed after PM permeabilization and reveals all AJs including aggregates within the cell. Cell clones are marked by the absence of GFP (blue). (**c**–**e**) Optical sections perpendicular to the apical–basal axis. (**c**′–**e**′) PM-labelled DE-cad channel alone. Clone border is marked by the dashed red line. (**c**) *RabX1* mutant cells accumulate endocytosed E-cadherin in the existing AJ aggregates (arrows). (**d**) *Rab11* cells show intracellular Arm accumulation (red) but no aggregation of endocytosed DE-cad within the cell. (**e**) *RabX1 Rab11* double-mutant cells accumulate endocytosed DE-cad in the existing AJ aggregates (arrows). (**f**–**h**) Optical sections along the apico-basal axis. (**f**) PM-labelled DE-cad localizes to the ZA (arrow) in *RabX1* mutant cells. (**g**,**h**) PM-labelled DE-cad localizes in *Rab11* single and *RabX1 Rab11* double-mutant cells to the apical PM (arrows). Scale bar, 10 μm.

A possible explanation for the concentration of Rab5 and Rab11 within the DE-cad aggregates in *RabX1* cells is a disruption of the recycling process at the level of early to recycling endosome conversion. To test directly whether DE-cad recycling is blocked in *RabX1* mutants we performed endocytosis assays with ovaries harbouring *RabX1* mutant cells. After 3-h incubation with the DE-cad antibody, we detected the protein. We also counterstained for Arm to visualize the intracellular aggregates. Notably, PM-labelled DE-cad was clearly detectable in the existing aggregates confirming that they accumulate endocytosed protein (Fig. 5c,c′ arrows; DE-cad Arm overlay was detectable in 31 of 32 clones).

We next tested the genetic hierarchy between *RabX1* and *Rab11* by generating *Rab11* clones in homozygous *RabX1* mutant epithelia. *RabX1 Rab11* double-mutant cells resemble *Rab11* single mutants as they form numerous small intracellular aggregates, which show a strong overlap with the Golgi (Fig. 5a,b; Supplementary Table 3). This indicates that DE-cad exocytosis to the ZA is disturbed like in *Rab11* single mutants.

To assess epistasis during DE-cad recycling we performed endocytosis assays in *RabX1* mutant ovaries with *Rab11* clones. Notably, double-mutant cells accumulated endocytosed DE-cad similar to *RabX1* single mutants (Fig. 5e,e′; DE-cad Arm overlay was detectable in 12 of 13 clones). Thus, *RabX1* acts upstream of *Rab11* in DE-cad recycling. This raises the possibility that RabX1 activates Rab11. We propose that in the absence of RabX1, inactive Rab11 and DE-cad are trapped together in a blocked recycling endosome. This provides an explanation for our observation that Rab11 and DE-cad co-accumulate in *RabX1* mutant cells (Fig. 4d).

### A cadherin flow localizes DE-cad to the ZA

Our DE-cad endocytosis experiments revealed that only a part of PM-labelled DE-cad accumulated within *RabX1* cells, whereas a significant amount was transported to the ZA (Fig. 5f; arrow). PM-labelled DE-cad in *RabX1 Rab11* double and in *Rab11* single-mutant cells even localized to the apical PM (Fig. 5g,h; arrows). This apical and apicolateral localization of PM-labelled DE-cad could be mediated by a *RabX1* and *Rab11*-independent transport mechanism. Such a recycling independent transport has been reported for mammalian cells and was named ‘Cadherin flow'. This flow was shown to move E-cad within the lateral PM in apical direction^41^.

**Figure 6:**
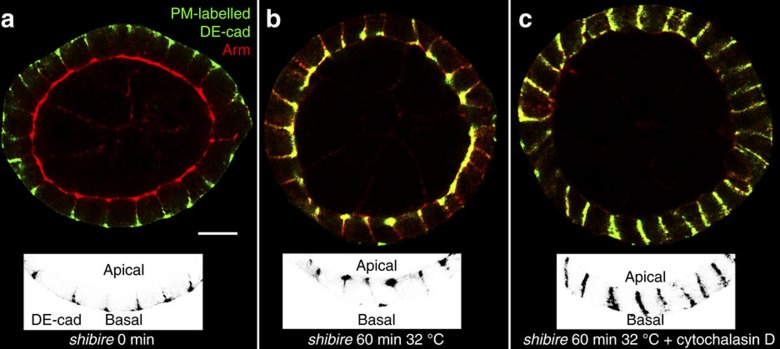
DE-cad is transported to the ZA by an actin-dependent Cadherin flow. Sagittal sections of egg chambers homozygous mutant for the temperature-sensitive *shibire*^*ts1*^ allele showing the apical–basal axis of the follicular epithelium. PM-labelled DE-cad is shown in green and total AJs are revealed by Arm counterstaining (red). Insets show the DE-cad channel of the lower part of the egg chamber. (**a**) Brief PM-labelling of DE-cad and immediate detection reveals the protein only at the basolateral PM. (**b**) Brief labelling, washing and subsequent incubation at the restrictive temperature for 60 min reveals an apically directed redistribution of DE-cad from the basolateral PM to the ZA. (**c**) Inhibition of actin polymerization by Cytochalasin D blocks directed redistribution and PM-labelled DE-cad localizes homogeneously along the lateral PM. Scale bar 10 μm.

To test whether a Cadherin flow exists in the follicular epithelium we analysed PM-labelled DE-cad distribution in the absence of endocytosis. We blocked endocytosis by using a temperature-sensitive mutation of *Dynamin* (*shibire*^*ts1*^), a critical regulator of endocytic vesicle scission^42^. Ovaries were briefly incubated with the antibody and basolateral localization of labelled DE-cad was confirmed (Fig. 6a). Strikingly, after 60 min at restrictive temperature almost all DE-cad accumulated at the ZA (Fig. 6b). Thus, lateral DE-cad is efficiently transported from the basal to the apical part of the lateral PM in absence of endocytosis. This indicates the existence of an apically directed DE-cad flow, which mediates ZA localization.

To characterize the dynamics of the DE-cad flow we measured the extent of the apical movement of basolaterally labelled DE-cad at different time points in *shibire* mutants. Within the first 8 min DE-cad reached the middle region of the PM indicating fast membrane flow in the basal half of the lateral PM (Supplementary Fig. 5). Interestingly, DE-cad reaches the ZA only after 30 min. This indicates that the DE-cad flow is slower in the apical half of the PM.

The Cadherin flow in mammalian cells is inhibited when actin filaments are disrupted^41^. We therefore performed DE-cad labelling experiments in *shibire* ovaries in the presence of cytochalasin D. F-actin disruption abolished the accumulation of DE-cad at the ZA resulting in homogenous distribution along the lateral PM (Fig. 6c). This indicates that the apically directed DE-cad flow is dependent on actin filaments. Thus, three mechanisms facilitate the accumulation of DE-cad at the ZA: transport of newly synthesized DE-cad, targeted recycling of endocytosed protein and an apically directed DE-cad flow within the lateral PM.

## Discussion

**Figure 7:**
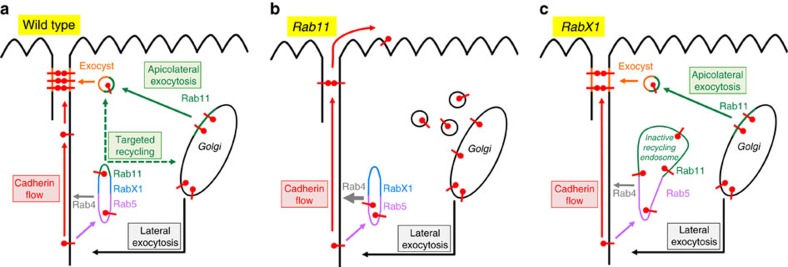
Model summarizing the mechanisms controlling DE-cad localization. Arrows within the cell show membrane trafficking routes of DE-cad. The red arrow outside of the cell indicates an apically directed flow within the lateral PM that is dependent on the actin cytoskeleton. Pathways analysed in this study are named in the boxes. Dashed arrows indicate that targeted DE-cad recycling might occur on a direct route from the recycling endosome to the ZA and/or on an indirect route including sorting in the Golgi. (**a**) Wild-type cell shows a tubular structure (Rab5, RabX1 and Rab11) representing the early endosome and recycling endosome, where endocytosed DE-cad is sorted for recycling. DE-cad is also directly recycled by Rab4 (grey arrow). (**b**) In *Rab11* mutant cells all endocytosed DE-cad is directly recycled by Rab4. Apicolateral exocytosis is abolished and DE-cad vesicles accumulate within the cell. Absence of apicolateral exocytosis prevents ZA maintenance. The membrane flow is no longer restricted by the ZA and transports DE-cad into the apical PM domain. (**c**) In *RabX1* mutant cells endocytosed DE-cad accumulates in a large Rab5 and Rab11 compartment, which is unable to mediate recycling. The presence of Rab11 in this compartment might prevent that all endocytosed DE-cad is recycled by Rab4. The ZA is only partially affected as apicolateral exocytosis and membrane flow are functional.

Our study reveals that vesicles with newly synthesized DE-cad leave the Golgi by two different pathways: lateral and apicolateral exocytosis (Fig. 7a). *Rab11* functionally distinguishes these two pathways as the gene is central for apicolateral but dispensable for lateral exocytosis. The lateral pathway could be common to all transmembrane proteins facilitating cell–cell adhesion including Fas2 and Fas3. Lateral adhesion proteins underlie a dynamic turnover. After internalization they are delivered to the early endosome in a *Rab5*-dependent process. Within the early endosome they are sorted to a large extent for return to the PM^8^,^11^,^43^. Commonly, recycling occurs either via direct recycling mediated by Rab4 or via the recycling endosome, which is regulated by Rab11 (ref. 18). It is likely that DE-cad is recycled by both Rab4- and Rab11-dependent mechanisms.

We identify *RabX1* as a critical new component for DE-cad recycling, and our data place its function in between the early and the recycling endosome. In *RabX1* mutants endocytosed DE-cad protein is not properly recycled but accumulates together with Rab5 and Rab11 in a large compartment (Fig. 7c). *RabX1* mutants are viable, which implies that recycling is not completely blocked, and consistent with this we observed a normal Rab4 distribution in *RabX1* mutants. It seems therefore likely that in *RabX1* mutants Rab11-mediated DE-cad recycling is blocked, while Rab4 mediated recycling is still functional.

Two compartments, the recycling endosome and the Golgi, provide DE-cad for ZA accumulation. It is unclear whether an interchange of DE-cad vesicles between these two compartments exists in the follicular epithelium. For instance it is possible that DE-cad, which leaves the Golgi is first transported to the recycling endosome, where it undergoes a sorting step for ZA delivery. However, the phenotypic differences between *RabX1* and *Rab11* mutant cells argue against this hypothesis. Our data indicate that *RabX1* mutants specifically affect DE-cad recycling, whereas *Rab11* mutants block apicolateral exocytosis of DE-cad but do not prevent recycling (probably because Rab11-mediated recycling is completely replaced by Rab4 mediated recycling). If newly synthesized DE-cad had to pass the recycling endosome, then it should accumulate there together with the endocytosed DE-cad in *RabX1* mutants, and this would lead to severe ZA defects. However, *RabX1* cells show only mild defects in comparison with *Rab11* cells arguing that *de novo* synthesized DE-cad does not pass the recycling endosome. It remains to be determined whether endocytosed DE-cad traffics to the Golgi for a sorting step for ZA delivery.

The exocyst complex is central for targeting DE-cad to the ZA. Biochemical studies indicate that Rab11 protein recruits the exocyst complex to DE-cad vesicles for their targeted fusion with the ZA^44^,^45^,^46^,^47^. It has been shown that in exocyst mutants endocytosed DE-cad does not traffic to the ZA^11^. Our analysis shows that exocyst mutant cells form, like Rab11 cells, numerous small AJ aggregates indicating defects in apicolateral exocytosis of newly synthesized DE-cad. Thus, exocyst mutants combine recycling and apicolateral exocytosis defects supporting the idea that the exocyst complex targets both vesicles with newly synthesized and with endocytosed DE-cad to the ZA.

Lateral DE-cad reaches the ZA not only by targeted recycling but also by an actin-dependent basal to apical DE-cad flow within the PM. Notably, after disruption of the flow, DE-cad spreads homogeneously within the lateral PM but neither enters the apical nor the basal PM domain (Fig. 6c). This indicates that the lateral PM is enclosed by barriers, which prevent the exit of DE-cad. The apical barrier is likely to be formed by the ZA itself. In *Rab11* mutant clones the ZA is not maintained and the membrane flow transports DE-cad into the apical domain (Fig. 7b and Fig. 5g, arrow). Interestingly, in *Rab11* cells neither the lateral marker Fas3 moves into the apical PM nor the apical marker aPKC redistributes into the lateral PM (Supplementary Fig. 6). This indicates that there is no free exchange between apical and lateral proteins in *Rab11* cells. Hence, the flow seems to be specific for DE-cad and does not transport other adhesion proteins.

Analysis of the cadherin flow in mammalian cells suggested that VE-cadherin is moving as a trans dimer in apical direction^41^. In our experiments, DE-cad was labelled with an antibody that binds to the cadherin domains^32^, which is likely to interfere with trans interactions. This suggests that trans interactions are no pre-requisite for the Cadherin flow in the follicular epithelium.

Interestingly, our analysis of the dynamics of DE-cad movement indicates that the flow is faster in the basal region of the lateral PM than in its apical part. This could be explained by different mechanisms driving the flow in the basal and apical regions of the lateral PM. It is also possible that there is more resistance in the apical part slowing down the velocity of the flow. Moreover, the actin cytoskeleton, which provides routes for the cadherin flow^41^ might be organized differently in basal and apical regions of the lateral PM.

The finding that various pathways control DE-cad distribution raises the question why E-cad localization underlies such complex regulation. The *Rab11* phenotype indicates that apicolateral exocytosis is essential for epithelial cell shape as mutant cells lose their ZA and flatten. E-cad localization to the lateral PM is important for the assembly of a contractile lateral actin cortex^7^. Interestingly, a balance of tension of the actin network of the ZA versus the actin network of lateral AJs has been identified in mammalian epithelial cells. This balance is important for epithelial integrity, and cells extrude from the epithelium when it is disturbed^7^. The ratio between apicolateral and lateral DE-cad exocytosis is a first step in regulating E-cad distribution between the lateral and apicolateral PM, which is likely to impinge on the tension balance.

The ratio between apicolateral and lateral DE-cad levels is also regulated by transporting lateral DE-cad to the ZA. This is mediated by targeted recycling and by the Cadherin flow. Analysis of *shibire* mutants shows that the flow is able to localize DE-cad efficiently in the absence of targeted recycling. This flow could be important for the establishment of the ZA during MET. In follicle stem cells and their mesenchymal precursors DE-cad localizes broadly along the lateral PM. Only after MET completion, DE-cad concentrates apicolaterally to establish the ZA^24^,^48^. This redistribution could be supported by the Cadherin flow. The maintenance of the ZA is facilitated by targeted recycling. When targeted recycling is blocked, like in *RabX1* mutants, cells show ZA and cell shape defects. This indicates that the DE-cad flow cannot fully compensate the loss of the targeted recycling pathway.

The possibility of differentially activating the various pathways controlling DE-cad localization provides an intriguing tool for epithelial cell shape changes. For instance, shutdown of lateral exocytosis in favour of enhanced apicolateral exocytosis could promote shrinking of the lateral PM and simultaneous expansion of the ZA, a process necessary to form squamous epithelia. By contrast, enhanced lateral exocytosis might support the expansion of the lateral PM when epithelial cells adopt a columnar shape. Thus, modulation of the activities of the different pathways could drive epithelial morphogenesis.

Disturbances of the pathways in differentiated epithelial tissues will compromise cell–cell adhesion and tissue architecture. It is for example tempting to speculate that loss of apicolateral exocytosis combined with an unrestricted Cadherin flow disturbs the organization of membrane domains in tumours, similar to our observations in *Rab11* cells. It will be important to explore how such misregulations contribute to disease processes like organ fibrosis and metastasis.

## Methods

### *Drosophila* genetics

Following stocks were used: *w; traffic jam*-Gal4 (ref. 37; provided by J. Bennecke), GR1-Gal4*/*TM3*Ser*^49^ (provided by S. Roth), w;; *Rab11*^*dFRT*^/TM3*Sb*^50^*, w;;* FRT5377*, Hrb98DE::GFP* (all provided by R.S. Cohen), *w; Rab5*^*2*^ FRT40A*/*CyO^51^ (provided by A. Guichet), *yw;;* UAS-YFP*-RabX1* (ref. 52), *yw; RabX1*^*KG06805*^*/*CyO*, w;* Df(2R)BSC661*/*SM6a*, Shibire*^*TS1*^ (ref. 53), (all from Bloomington *Drosophila* Stock Centre), w; FRTG13 *sec6*^*Ex15*^/CyO^54^ (provided by Y. Bellaiche), *Rab5* RNAi (Vienna *Drosophila* Resource Center (VDRC) KK 103945 and VDRC GD 34096), *Rab11* RNAi (TRiP library, VALIUM10, BDSC), UAS-HA-*Rab11* (insertions on second and third chromosome), UAS-HA-*Rab5* (insertions on second and third chromosome), *w;* neoFRT42D *RabX1*^*KG06805*^, *hs-FLP; rab5*^*2*^ FRT40A *RabX1*^*KG06805*^*/*CyO*, w;* GFP FRT40A *RabX1*^*KG06805*^/CyO*, w;;*GR1-*Gal4* UAS-FLP/TM3*Ser* (all from this work).

*Genotypes of females generated for genetic epistasis studies*. Figure 1c: hs-FLP*; rab5*^*2*^ FRT40A*/GFP* FRT40A; *Rab11*^*dFRT*^*/*FRT5377*, Hrb98DE::*GFP

Figure 4a: hs-FLP; rab5^2^ FRT40A RabX1^KG06805^/GFP FRT40A RabX1^KG06805^

Figure 4b: *w;* FRT42D *RabX1*^*KG06805*^/FRT42D *GFP*; GR1-Gal4 UAS-FLP/UAS*-Rab5*-RNAi.

Figure 5a,b,e: hs-FLP; FRT42D RabX1^KG06805^/FRT42D RabX1^KG06805^; Rab11^dFRT^/FRT5377, Hrb98DE::GFP.

Supplementary Figure 1g: *w; traffic jam*-Gal4*/*UAS*-Rab5*-RNAi; UAS*-Rab11*-RNAi.

*Generation of a FRT42D RabX1^KG06805^ stock*. We first removed an unrelated P-element insertion on the first chromosome of the original stock. The second chromosome carrying the insertion in the RabX1 locus harboured an additional unrelated lethal mutation, which was removed by recombining the P-element in *RabX1* with neoFRT42D.

*Genotypes of females generated for rescue experiments*. Supplementary Fig. 3c,e: *w;* FRT42D *RabX1*^*KG06805*^/FRT42D GFP; GR1-Gal4 UAS-FLP*/*TM3*Ser*

Supplementary Figure 3d,f,: *w;* FRT42D *RabX1*^*KG06805*^/FRT42D GFP; GR1-Gal4 UAS-FLP*/*UAS**-YFP*-RabX1.*

### Generation of genetic mosaics and RNAi induction

Genetic mosaics were generated by using either a heat-shock (hs) or a UAS/Gal4 inducible Flippase (FLP). To induce the hs-FLP for homozygous mutant cell clones heat shocks were applied during pupal stages by placing the vials in 37 °C water bath for 1 h once a day until hatching. Females were dissected 24 to 48 h after hatching.

UAS-FLP was induced in follicular epithelium using a GR1-Gal4 UAS-FLP recombinant chromosome. Crosses with this chromosome were raised at room temperature to avoid excessive clone induction.

For RNAi knockdowns in the follicular epithelium UAS-inducible RNAi transgenes were driven by either *traffic jam*-Gal4 or GR1-Gal4. Crosses were raised at room temperature.

### Immunohistology

Ovaries were dissected in Schneider's medium (Gibco LifeTechnologies) and separated in their anterior part using fine needles. After fixation in 4% paraformaldehyde in PBS for 10 min. at room temperature, ovaries were washed and permeabilized with 0.1% TritonX-100 in PBS. All primary antibodies were diluted in 0.1% Triton/PBS and incubated for 3 h at room temperature. Secondary antibodies were incubated for 2 h. Ovaries were then stained with 0.5 μg ml^−1^ DAPI for 5 min, washed and mounted in Vectashield (Vector labs).

Primary antibodies were used at the following concentrations: mouse anti-Armadillo 1:100 (DSHB, clone N2 7A1, concentrate), rat anti-DE-cadherin 1:50 (DSHB, clone DCAD2, concentrate), mouse anti-Discs large 1:100 (DSHB, clone 4F3, concentrate), goat anti-GFP-FITC 1:200 (GeneTex, GTX26662), mouse anti-Fas3 1:200 (DSHB, clone 7G10, concentrate), mouse anti-Fas2 1:200 (DSHB, clone1D4, concentrate), rabbit anti-GM130 1:100 (Abcam, ab30637), guinea pig anti-Sec6 1:1,000 (provided by U. Tepass^47^), mouse anti-Rab11 1:100 (BD, catalogue number 610657); rabbit anti-Rab5 1:250 (Abcam, ab31261), rabbit anti-aPKC 1:200 (Santa Cruz Biotechnologies, cs-216), mouse anti-HA 1:100 (Santa Cruz Biotechnologies, sc-7379), rat anti-HA 1:100 (Roche, clone 3F10, catalogue number 1867423), rabbit anti-Rab4 1:200 (Abcam, ab78970). Alexa fluorophores coupled secondary antibodies (Invitrogen) were used in a 1:200 dilution.

Images were acquired using Leica LSM SP5 DS and SP5 MP confocal microscopes using × 63 oil immersion magnification at a resolution of 1,024 × 1,024 then adjusted for gamma, edited and assembled with Adobe Photoshop CS3.

### Analysis of DE-cad distribution in living ovaries

For the analysis of lateral DE-cad exocytosis living ovaries were dissected in Schneiders medium and ovarioles separated in their anterior part with fine insect needles. Subsequently, ovaries were incubated in DE-cad solution (DE-cad antibody (1:50) in Schneider's medium containing 10% fetal calf serum, 100 μg ml^−1^ bovine insulin (Sigma)) for 10 min at room temperature. After washing, ovaries were fixed and permeabilized followed by incubation with anti-Arm (Fig. 2a) or anti-Dlg (Fig. 2b) and anti-rat Alexa488 (to detect DE-cad) for two hours. The ovaries were washed and incubated with anti-mouse Alexa568 antibody to detect Arm or Dlg.

To follow DE-cad endocytosis and redistribution living ovaries harbouring *Rab11* or *RabX1* single or *RabX1 Rab11* double-mutant clones (Figs 1d and 5c–e) were continuously incubated in DE-cad solution for 3 h, then washed, fixed and permeabilized. Subsequently, ovaries were incubated with anti-GFP-FITC (to label the clones), anti-GM130 (Fig. 1d) or anti-Arm (Fig. 5c–e) and anti-rat Alexa568 antibodies (to detect DE-cad) for 2 h. Then ovaries were washed and incubated with anti-rabbit Alexa647 (for GM130) or chicken anti-mouse Alexa647 (for Arm) antibodies.

To analyse DE-cad distribution in the absence of endocytosis (Fig. 6a,b) *shibire*^*TS1*^ homozygous mutant flies were raised at 18 °C. Ovaries were dissected and opened in Schneider's medium and preincubated in a water bath at restrictive temperature (32 °C) for 20 min. Then DE-cad incubation was performed for 2 min using prewarmed (32 °C) DE-cad solution at 32 °C. After washing, ovaries were incubated for 60 min in prewarmed incubation solution (Schneider's medium containing 10% fetal calf serum, 100 μg ml^−1^ Insulin) at 32 °C to allow DE-cad redistribution. After washing, fixation and permeabilization ovaries were incubated with anti-Arm and anti-rat Alexa488 (to detect DE-cad) antibodies for 2 h. The ovaries were then washed and incubated with anti-mouse Alexa568.

To analyse the movement of PM-labelled DE-cad along the lateral membrane DE-cad (Supplementary Fig. 5) incubation was performed for 2 min in prewarmed DE-cad solution at 32 °C. After washing, ovaries were incubated for 1, 3, 5, 8, 12, 19, 25 and 30 min in incubation solution at 32 °C to allow DE-cad redistribution. After washing, fixation and permeabilization, DE-cad was detected and Arm counterstaining performed (see above). After image acquisition the membrane length from five randomly selected cells was measured. The distance between the most apical Arm signal and the most basal DE-cad signal revealed the total lateral PM length. Subsequently, the length of DE-cad signal along the lateral membrane was measured. The percentages of DE-cad signal along the total membrane lengths was determined and plotted against the incubation times.

To analyse DE-cad distribution when actin polymerization is inhibited (Fig. 6c) ovaries were preincubated with 100 μg ml^−1^ cytochalasin D (Sigma) in incubation solution for 20 min at restrictive temperature. Subsequently, ovaries were incubated for 2 min in DE-cad solution. After washing, the ovaries were incubated in incubation solution containing cytochalasin D in a final concentration of 100 μg ml^−1^ at 32 °C for 60 min. After washing, fixation and permeabilization, DE-cad was detected and Arm counterstaining performed (see above).

### Generation of transgenes

HA-Rab11 and HA-Rab5 transgenic flies were generated by cloning the open reading frame into the pUASp2 vector. Germline transformation was performed after standard procedure. cDNAs were obtained from *Drosophila* Genomics Resource Center (GM06568 for Rab11 and GH24702 for Rab5). cDNAs were amplified using the following primers: 5′- TGGATCCATGGGTGCAAGAGAAGACG -3′ 5′- GTCTAGATCACTGACAGCACTGTTTGCG -3′ (Rab11) and 5′- CGAATTCATGGCAACCACTCC -3′ 5′- TTTCTAGATCACTTGCAGCAGTTGTTCG -3′ (Rab5). The PCR product was cloned (BamHI and XbaI for Rab11 and EcoRI and XbaI for Rab5) into HA-tag carrying pUASp2 vector and sequenced resulting in Rab proteins with an N-terminal HA-tag.

### Image analysis

*Quantification of vertices and cell areas*. To count vertices and measure cell areas we developed a MATLAB script (Version: 8.5.0.197613 (MathWorks, R2015a)), which uses standard image process functions to highlight the cell boundaries on binary images. After boundaries recognition, the vertices were counted using the connectivity with the neighbour cells. The cells at the edges of the images were removed from the analysis. We only considered cells with a maximum of 8 vertices. We calculated the cell area using the ‘regionprops' MATLAB function.

*Quantification of vesicle co-localization*. To identify co-localizing vesicles ImageJ/Fiji script was developed. The source images are segmented using a low sigma Gaussian filter to highlight the vesicles in the green and in the red channel. Thresholds were applied to both channels to detect the vesicles. For each channel a binary image was created, and to separate fused vesicles the ‘erode' function was applied. After vesicle detection, the ‘analyse particles' tool was used to count the number of vesicles in the green and in the red channel. Overlapping vesicles were highlighted in yellow using the ‘image calculator' plugin and counted using the ‘analyse particles' tool. The batch mode was set on true to reduce RAM usage and to speed up the image analysis. To preferentially restrict the analysis to the vesicles, a minimum and maximum vesicle size area was set. Moreover, the circularity of the particles was considered for the analysis of some of the images.

*Quantification of Arm aggregates and Golgi overlap*. An ImageJ/Fiji script was developed to analyse the overlapping area between the Golgi and Arm aggregates. The source images were segmented using adapted Gaussian filters to highlight the Golgi and Arm signal and to reduce the image background. Thresholds were applied to detect the Golgi and Arm aggregates and for each channel a binary image was created. To count and measure the area of the Golgi and Arm aggregates the ‘analyse particles' tool was used. The overlapping regions were detected using the ‘*image calculator*' plugin, and the co-localizing areas were measured using the ‘analyse particles' tool. To restrict the analysis to the mutant cell clones a manual approach was used to remove detected objects form wild-type cells. The batch mode was set on true to reduce RAM usage and to speed up the image analysis. To restrict the detection to the Golgi and to Arm aggregates minimum and maximum size area was applied to both channels during the image analysis.

## Additional information

**How to cite this article:** Woichansky, I. *et al*. Three mechanisms control E-cadherin localization to the zonula adherens. *Nat. Commun.* 7:10834 doi: 10.1038/ncomms10834 (2016).

## Supplementary Material

Supplementary Information Supplementary Figures 1-6 and Supplementary Tables 1-3

## Figures and Tables

**Table 1:** Quantification of cell-shape defects in *RabX1* homozygous mutants.

**Genotype**	***n***	**4 vertices**	**5 vertices**	**6 vertices**	**7 vertices**	**8 vertices**	**Average cell area**
Wild type	196	2 (±0.7)**1%**	46 (±4.4)**23%**	116 (±5.2)**59%**	32 (±1.9)**16%**	0 (±0)**0%**	35.7 μm(±11.57)(*n*=196)
*RabX1*	345	23 (±2.3)**7%**	105 (±4.6)**30%**	134 (±5.6)**39%**	73 (±2.6)**21%**	10 (±1)**3%**	51.25 μm(±23.66)(*n*=352)

Optical confocal sections perpendicular to the anterior posterior axis of Arm-stained epithelia with the indicated genotype were analysed. Number of vertices of individual cells was determined and the area of each cell was measured as described in Methods. ±values represent s.d. from six independent experiments for the wild-type and 10 experiments for *RabX1* mutants.
