# Supplementary material for: Three mechanisms control E-cadherin localization to the zonula adherens
Source: Nat Commun. 2016 Mar 10;7:10834. doi: 10.1038/ncomms10834 (PMC4792928; doi:10.1038/ncomms10834)
Supplement: Supplementary Information — Supplementary Figures 1-6 and Supplementary Tables 1-3 [file ncomms10834-s1.pdf]

Supplementary Figures and Tables

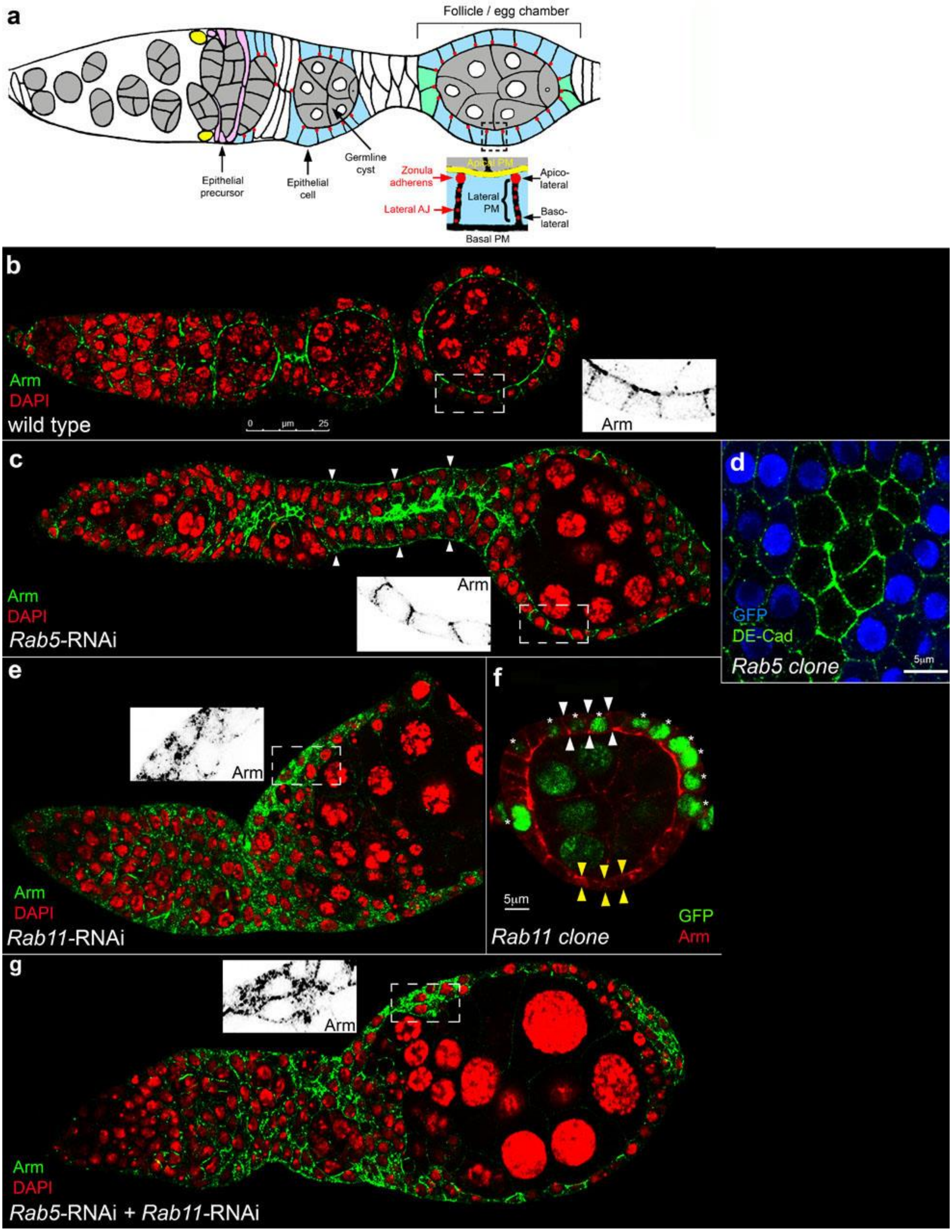

Supplementary Figure 1: Egg chamber formation and Arm localisation after *Rab5* and *Rab11* single and *Rab5 Rab11* double RNAi. (**a**) Egg chamber formation in the *Drosophila*

ovary. Germline cysts are depicted in grey. Stem cells for the follicular epithelium (yellow) divide to produce precursors (pink) that migrate towards the cysts. Polarisation of epithelial cells is reflected by AJs localisation to the sites where two epithelial precursors contact the germline cyst (red dots). Epithelial cells (blue) form an egg chamber by assembling a monolayer around the cyst. Follicle cell precursors differentiate also into stalk cells (white), which form cellular connections between egg chambers. (**b - e**) Germaria and early egg chambers stained for Arm (green). Insets show the Arm channel of the region marked by the white dashed box. (**b**) Arm localises in the wild type to the lateral PM and accumulates at the ZA. Note that some epithelial cells are cut at the level of the ZA, which is then visible as a continuous line. (**c**) Knockdown of *Rab5* (induced by *traffic jam*-Gal4) disrupts Arm accumulation at the ZA and leads to homogeneous Arm distribution at the cell periphery. Stalk cell number is dramatically increased (arrowheads). **(d**) *Rab5* mutant cell clone (marked by the absence of GFP (blue)) shown in an optical section perpendicular to the apico-basal axis of the follicular epithelium. Mutant cells accumulate DE-cad at the PM. (**e**) Knockdown of *Rab11* (induced by *traffic jam*-Gal4) results in cytoplasmic Arm accumulation. Moreover, formation of a monolayered epithelium and stalk generation is abolished. (**f**) Egg chamber with *Rab11* mutant cell clones (marked by the absence of GFP (green)) and highlighted by asterisks. Within the big clone in the lower part (yellow arrowheads) cells do not form a ZA, lose their cuboidal shape and become flat (comparing the distances between the white and yellow arrowheads).. Note that the small clones in the upper part retain their ZA and stay cuboidal. (**g**) Simultaneous RNAi of *Rab5* and *Rab11* (induced by *traffic jam*-Gal4) leads to Arm accumulation within the follicle cells and disrupts stalk and epithelial formation like the *Rab11* single knockdown.

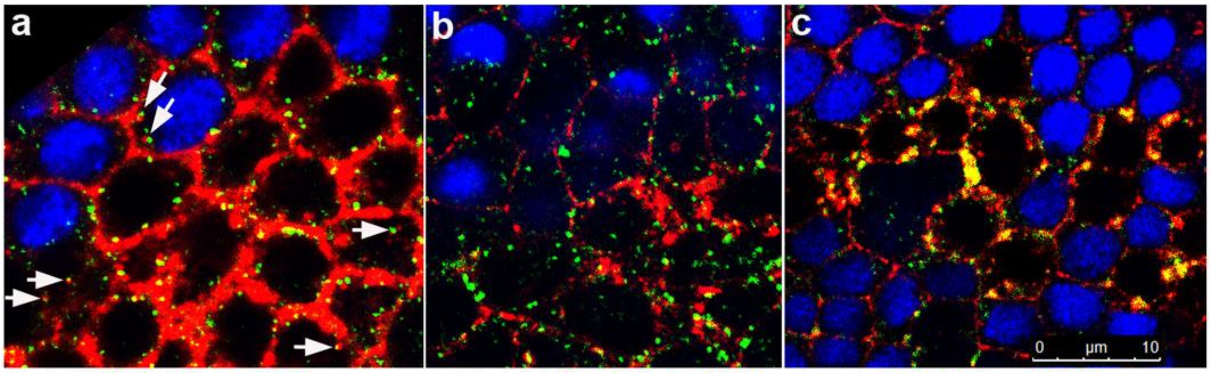

Rab11 Rab4 Arm GFP *Rab11* Sec6 Arm GFP *Sec6* Rab11 Arm GFP

Supplementary Figure 2: Intracellular DE-cad aggregates in Rab5, Rab11 and*Sec6* mutant cells. Mutant cell clones (marked by the absence of GFP (blue)) are shown in optical sections perpendicular to the apico-basal axis of the follicular epithelium. (**a**) Rab4 puncta (green) partially overlap with or are in close proximity to Arm puncta within wild type and *Rab11* mutant cells (arrows). (**b**) The Arm aggregates (red) in *Rab11* mutant cells show almost no overlap with Sec6 protein (green). (**c**) Clones mutant for the exocyst component Sec6 form numerous small AJ aggregates within the cell (Arm staining in red). These aggregates also accumulate Rab11 protein (green).

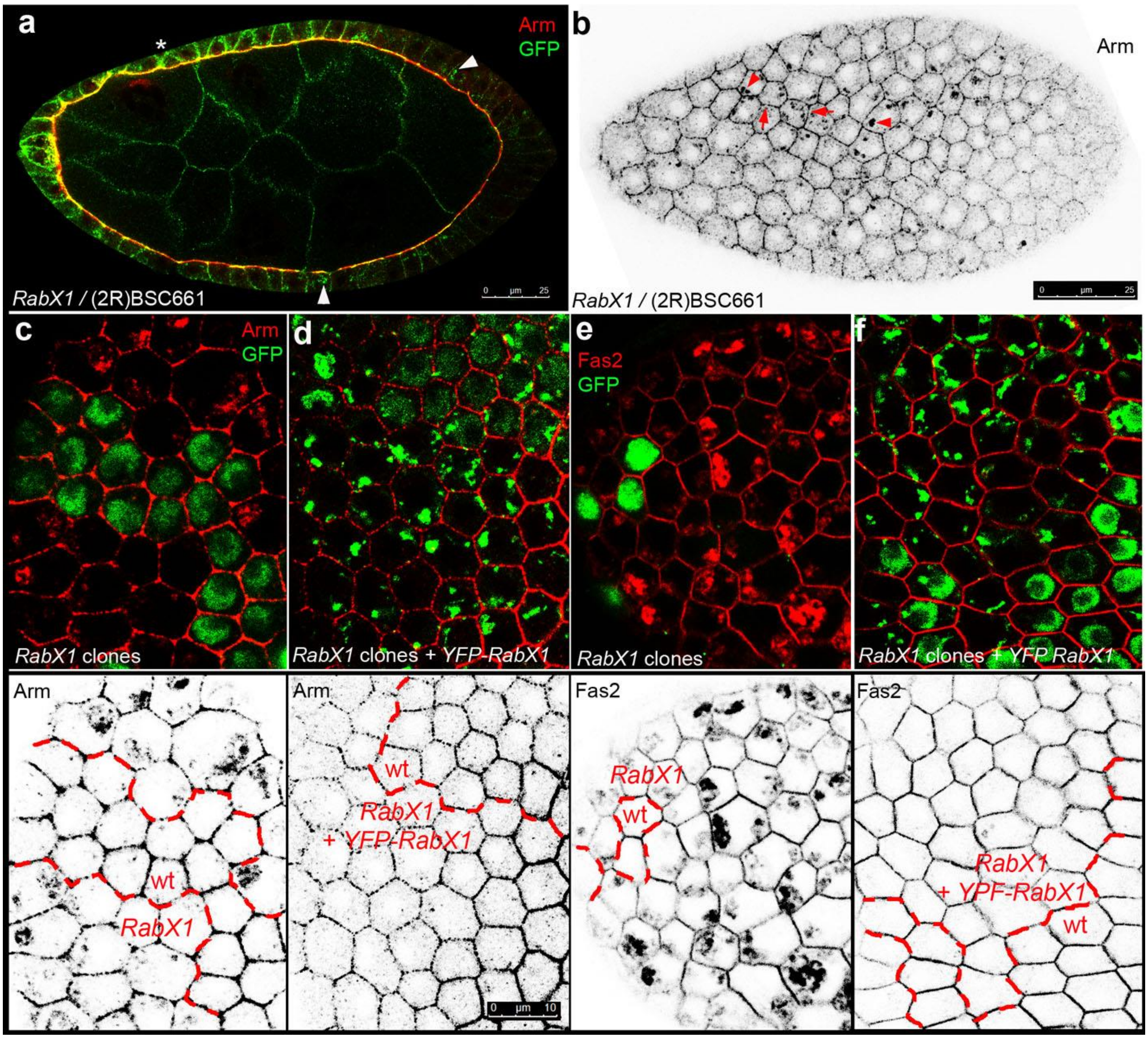

Supplementary Figure 3: ***RabX1*^{KG06805} is a strong allele whose phenotype is rescued by the expression of YFP-*RabX1*.** (**a, b**) Egg chambers transheterozygous for *RabX1*^{KG06805} and Df(2R)BSC661, a deficiency uncovering the *RabX1* locus. (**a**) Sagittal section showing aPKC (red) and Arm (green) localisation. Epithelial cells show shape defects (asterisk) and intracellular Arm accumulation (arrowhead) like homozygous *RabX1*^{KG06805} mutants. (**b**) Optical section perpendicular to the apical-basal axis of the epithelium showing the distribution of Arm. AJ aggregates are marked by arrowheads and gaps in the ZA by arrows. (**c - f**) Rescue of protein aggregation in *RabX1* mutant cell clones by the expression of YFP-*RabX1*. Epithelia harbouring *RabX1* mutant cell clones, which are marked by the absence of GFP (diffuse green) are shown in sections perpendicular to their apical-basal axis. Clones were induced by a UAS-Flippase transgene whose expression was driven by GR1-Gal4. In the epithelia in (**d** and **f**) GR1-Gal4 simultaneously induced the

expression of a UAS-YFP-*RabX1* transgene to rescue the *RabX1* phenotype. The egg chambers shown in (**c** and **d**) and in (**e** and **f**) are siblings from the same cross and were stained in parallel. (**c**) Cell clones depicting the intracellular accumulation of Arm in *RabX1* clones. Clone border is marked by the dashed line. (**d**) Expression of YFP-*RabX1* in *RabX1* mutant cells. GFP staining detects a diffuse signal marking the wild type cells and an intense signal identifying YFP-RabX1 protein. *RabX1* mutant cells have lost the diffuse cytoplasmic GFP staining but retain the intense green signal. Lower panel depicts Arm channel alone. *RabX1* mutant cells do not form Arm aggregates when YFP-RabX1 is expressed. (**e**) *RabX1* cell clone revealing Fas2 aggregates. (**f**) Expression of YFP-*RabX1* rescues the intracellular Fas2 accumulation. Lower panels show Fas2 channel alone.

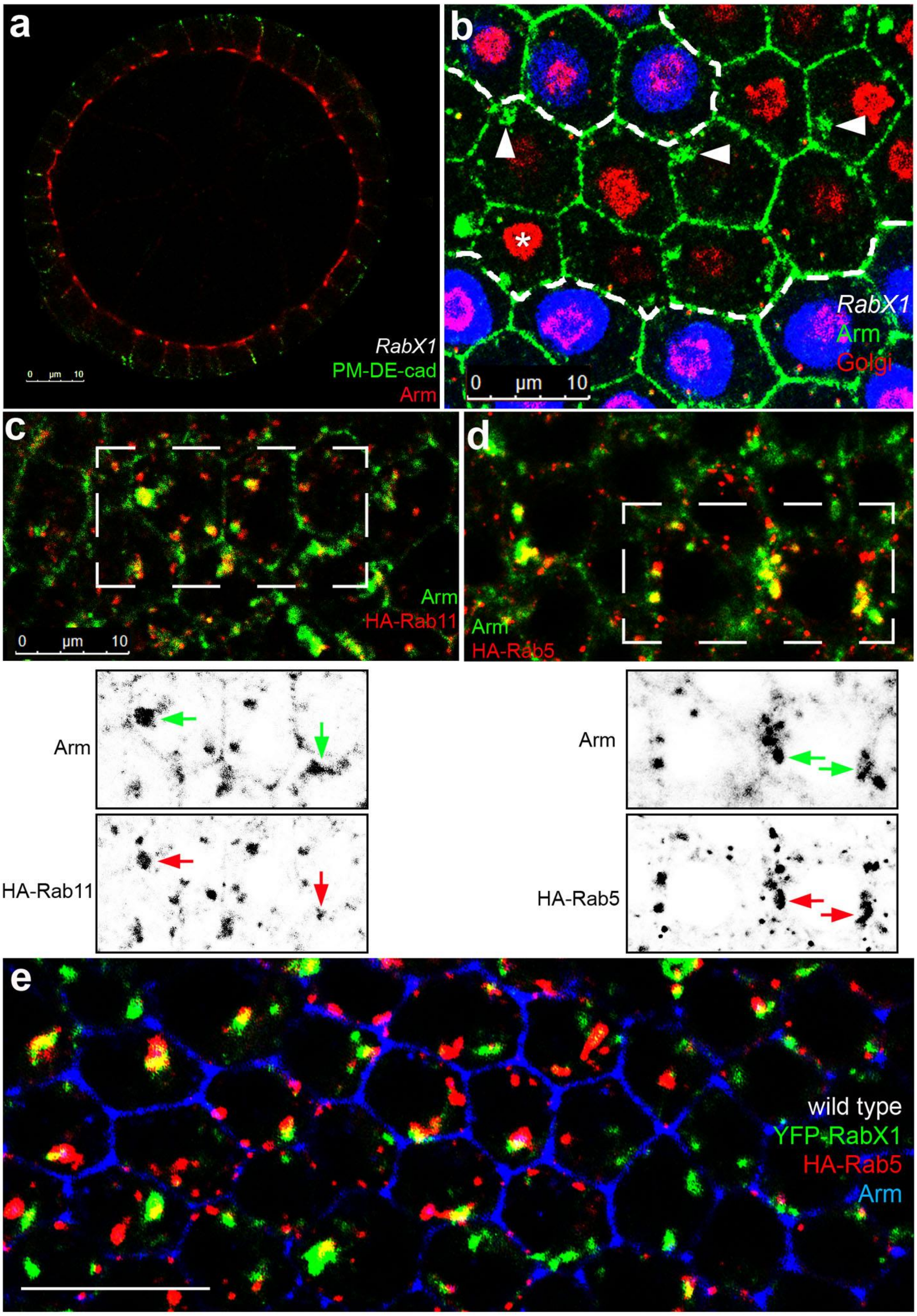

Supplementary Figure 4: **Analysis of *RabX1* mutants and RabX1 localisation.** (**a**) *RabX1* mutant egg chamber, which was fixed directly after antibody incubation. DE-cad is detectable at the basolateral PM indicating normal lateral exocytosis. (**b**) *RabX1* clones marked by the absence of GFP. Arrowheads indicate Arm (green) aggregates, which are not connected with the Golgi (red, detected by GM130 staining). The asterisk highlights

unspecific nuclear GM130 staining. (**c, d**) Homozygous mutant *RabX1* epithelia are shown in optical sections perpendicular to their apical-basal axis. Lower panels show single channels of the region marked by the dashed box. (**c**) GR1-Gal4 induced HA-Rab11 (red) accumulates within the Arm aggregates (green) in *RabX1* mutants. (**d**) GR1-Gal4 induced HA-Rab5 (red) accumulates within the Arm aggregates (green) in *RabX1* mutants. (**e**) *traffic jam*-Gal4 driven YPF-RabX1 (green) and HA-Rab5 (red) proteins overlap (arrowheads).

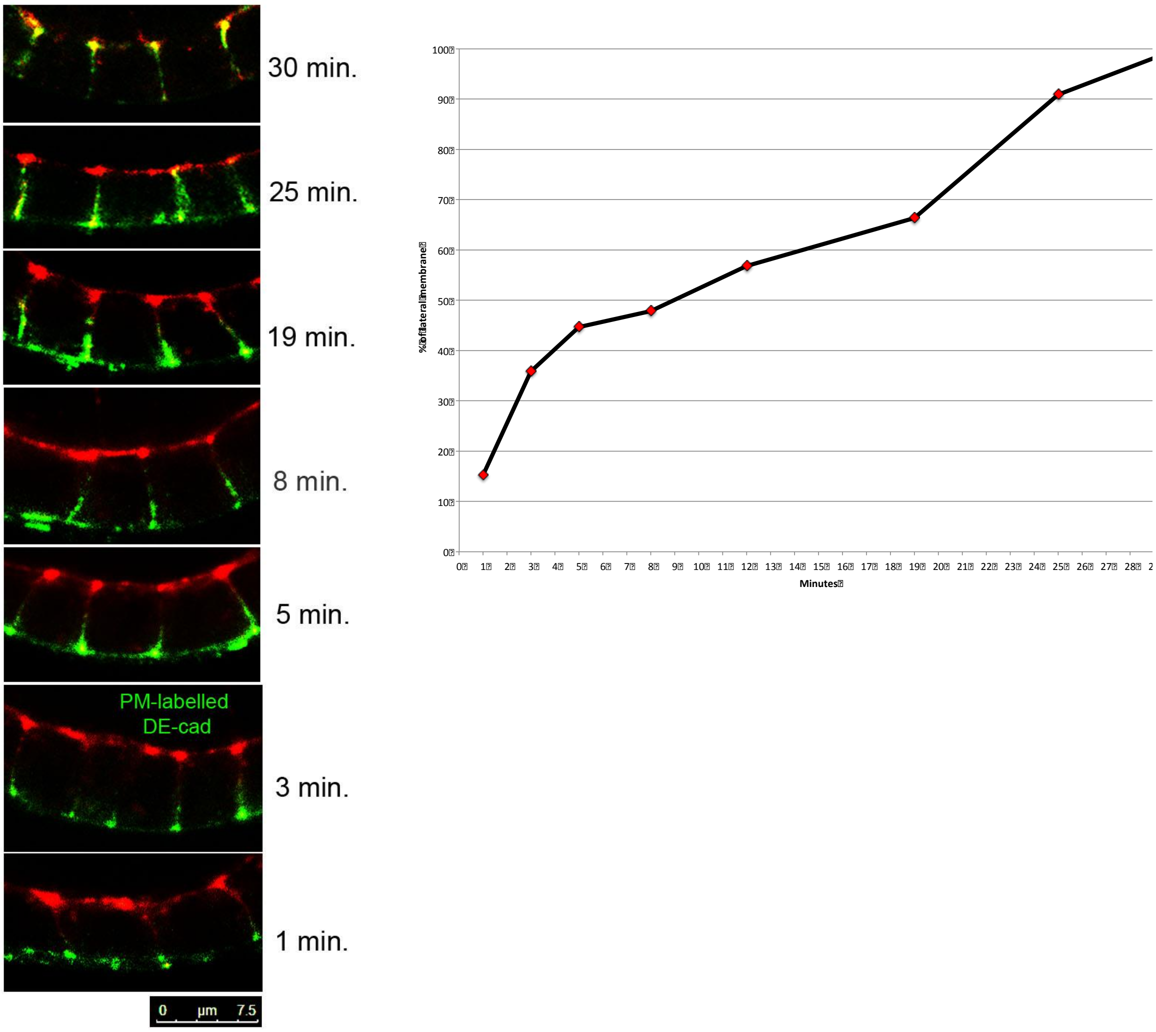

Supplementary Figure 5: (**a,** left) **Analysis of membrane flow dynamics in *shibire* mutants.** *shibire* mutant ovaries were briefly incubated with the anti-DE-cad antibody and samples were taken at the indicated time points to determine how far DE-cad protein (green) was transported in apical direction. Ovaries were counterstained for Arm (red) to detect the ZA. Three representative epithelial cells of an egg chamber are shown. (**b,** right) Graph summaries how far the DE-cad signal spread in apical direction along the lateral PM at the indicated time points (red rhombi).

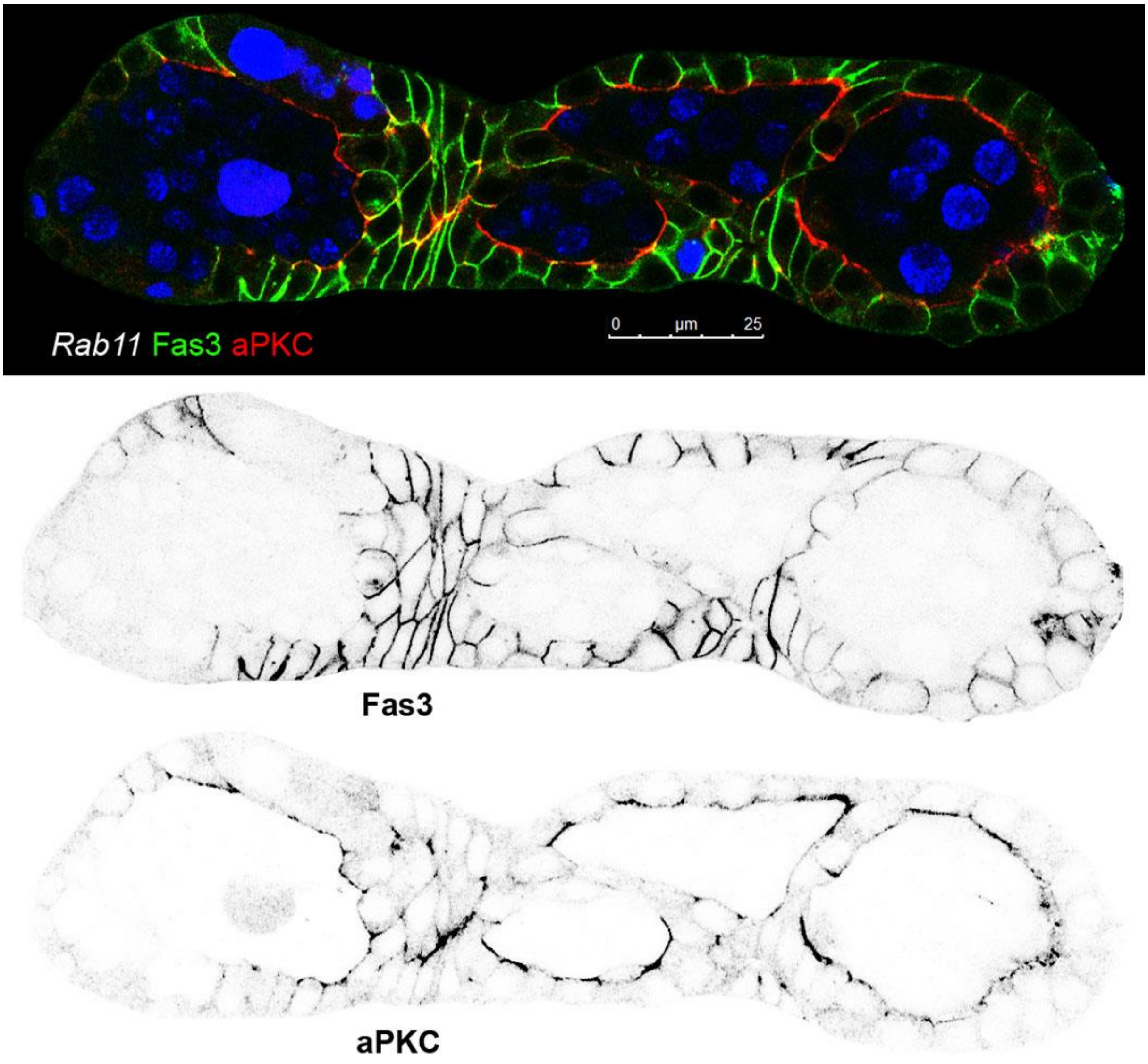

Supplementary Figure 6: Fas 3 and aPKC stay within their membrane domains after loss of *Rab11*. Ovariole with follicle stem cell clones mutant for *Rab11* is depicted in a sagittal confocal section. Absence of *Rab11* in the epithelial cells is indicated by the loss of GFP (blue). Cells of the inner germline cyst retain Rab11. Both the lateral marker Fas3 (green) and the apical marker aPKC stay in their domain indicating global maintenance of membrane polarity. Lower panels show Fa3 and aPKC channels alone.

Figure	Genotype (Staining)	Number of analysed egg chambers/ ovarioles	Number of egg chambers/ ovarioles with described phenotype	Number of analysed pictures	Number of Experiments
Fig. 2a	wt, 0 min. (PM-labelled DE-cad, Arm)	7	7	7	1
Fig. 2b	*Rab11* RNAi	13	13	13	2
Fig. 3d,f	*RabX1*(Arm)	111	109	111	11
Fig. 6a	*shibire*^{ts1} 32°C, 0 min. (PM-labelled DE-cad, Arm)	18	18	18	2
Fig. 6b	*shibire*^{ts1} 32°C, 60 min. (PM-labelled DE-cad, Arm)	18	18	18	2
Fig. 6c	*shibire*^{ts1} 32°C, + Cytochalasin D, 60 min. (PM-labelled DE-cad, Arm)	16	16	16	2
Supplementary Fig. 1c	*Rab5* RNAi (Arm)	25	25	25	3
Supplementary Fig. 1d	*Rab11* RNAi (Arm)	14	14	14	3
Supplementary Fig. 1e	*Rab5, Rab11* double RNAi (Arm)	9	9	9	2
Supplementary Fig. 3a,b	*RabX1*/(2R)BSC661 (Arm)	23	23	23	4
Supplementary Fig. 4a	*RabX1,* 0 min. (DE-Cad, Arm)	8	8	8	2
Supplementary Fig. 4c	*RabX1*, HA-*Rab11*- (Arm)	12	11	12	1
Supplementary Fig. 4d	*RabX1*, HA-*Rab5* (Arm)	15	14	15	1
Supplementary Fig. 5	*shibire*^{ts1} 32°C, 0-30 min. (PM-labelled DE-cad, Arm)	94	92	94	1

Supplementary Table 1: **Summary of results of experiments performed in wild type and homozygous mutant flies and in flies in which RNAi was induced in ovaries.**

Figure	Genotype (staining)	Number of clones analysed	Number of clones with described phenotype	Number of analysed pictures	Number of experiments
Fig. 1a	*Rab5* clones (Arm)	32	31	18	2
Fig. 1b	*Rab11* clones (Arm)	94	87	70	5
Fig. 1c	*Rab5 Rab11* double (Arm)	18	18	19	4
Fig. 1d	*Rab11* clones (PM-labelled DE-Cad + GM130)	18	17	20	2
Fig. 1e	*Rab11* clones (DE-Cad + GM130)	17	16	2	2
Fig. 1f	*Rab11* clones (Arm + GM130)	41	34	35	3
Fig. 2c	*Rab11* clones (Fas3)	26	25	21	2
Fig. 2d	*Rab11* clones (Fas2)	19	18	15	2
Fig. 3b	*RabX1 clones (DE-cad +Fas2)*	6	6	5	1
Fig. 4a	*Rab5* clones in *RabX1* (Arm)	34	33	27	2
Fig. 4b	*Rab5* RNAi in *RabX1* clones (Arm)	15	15	16	1
Fig. 4c	*RabX1 clones* (Arm + Rab5)	7	7	5	1
Fig. 4d	*RabX1 clones* (Arm + Rab11)	6	6	6	1
Fig. 5a	*Rab11* clones in *RabX1* (Arm)	59	51	89	3
Fig. 5b	*Rab11* clones in *RabX1* (Arm + GM130)	27	20	22	3
Fig. 5c	*RabX1 clones* (PM-labelled DE-Cad + Arm)	32	31	25	3
Fig. 5d	*Rab11 clones* (PM-labelled DE-Cad + Arm)	25	24	9	1
Fig. 5e	*Rab11 clones in RabX1* (PM-labelled DE-Cad + Arm)	13	12	9	2
Supplementary Fig. 1d	*Rab5* clones (DE-cad)	19	18	13	2
Supplementary Fig. 2a	*Rab11* clones (Rab4 + Arm)	10	9	8	2
Supplementary Fig. 2b	*Rab11* clones (Sec6 + Arm)	7	6	6	1
Supplementary Fig. 2c	*Sec6* clones (Rab11 + Arm)	12	12	15	1
Supplementary Fig. 3d	*RabX1* clones + YFP-*RabX1* (GFP + Arm)	18	18	20	1
Supplementary Fig. 3f	*RabX1* clones + YFP-*RabX1* (GFP + Fas2)	8	8	7	1
Supplementary Fig. 4b	*RabX1* clones (Arm + GM130)	23	23	24	2
Supplementary Fig. 6	*Rab11 clones (Fas3 + aPKC)*	23	19	15	1

Supplementary Table 2: **Summary of results from experiments performed in genetic mosaic flies**

1<br>Genotype	2<br>Number of Arm aggregates per cell	3<br>Number of Golgi-Arm overlaps per cell	4<br>Average size of Arm aggregates in μm^2
Rab11<br>(n=53*)*	**4.8**<br>(+/- 0.58)	**2.7**<br>(+/- 1.36)	**0.7**<br>(+/- 0.37)
RabX1<br>(n=54)	**1.4**<br>(+/- 0.09)	**0.4**<br>(+/- 0.05)	**1.6**<br>(+/- 0.46)
RabX1 +<br>*Rab11* (n=47)	**4.7**<br>(+/- 0.41)	**6.2**<br>(+/- 0.16)	**2.1**<br>(+/- 0.38)

Supplementary Table 3: Quantification of Arm aggregation and Arm Golgi overlap. Analysis of cell clones mutant for the indicted genes stained for Arm and the Golgi marker GM130. Images of confocal sections perpendicular to the apical-basal axis were quantified (see material and methods). Colum 2 shows the total number of areas with delimited cytoplasmic Arm signal (Arm aggregates) divided by the number of analysed cells. Colum 3 shows how often these areas overlap with the Golgi signal. The average size of a delimited area with Arm signal is shown in column 4. Quantification was restricted to the mutant cells. Note that the values refer only to one confocal section but not to the whole cell volume. +/- values represent s.d. from two independent experiments for Rab11 single and RabX1 Rab11 double mutants and three experiments for RabX1 single mutants.
